# Enhancement of photovoltage by electronic structure evolution in multiferroic Mn-doped BiFeO_3_ thin films

**DOI:** 10.1038/s41598-020-71928-5

**Published:** 2020-09-21

**Authors:** Seiji Nakashima, Tohru Higuchi, Akira Yasui, Toyohiko Kinoshita, Masaru Shimizu, Hironori Fujisawa

**Affiliations:** 1 Department of Electronics and Computer Science, Graduate School of Engineering, University of Hyogo, Himeji, Hyogo 671-2201 Japan; 2 Department of Applied Physics, Tokyo University of Science, Katsushika, Tokyo 125-8585 Japan; 3 Japan Synchrotron Radiation Research Institute / SPring-8, Sayo, Hyogo 679-5198 Japan

**Keywords:** Solar cells, Electrical and electronic engineering

## Abstract

The bulk photovoltaic effect (BPVE) is a mechanism of recent focus for novel solar cells that exceed the power conversion efficiency of p–n junction solar cells because of the quantum mechanical effect to generate photocurrent known as shift current. Ferroelectrics are receiving attention again because of their high voltage generation by the BPVE and converse piezoelectric effect to realize high performance optical actuators. We have investigated the BPVE in ferroelectric BiFeO_3_ (BFO) single crystal thin films, whereby the photovoltage was enhanced by Mn doping, and 852 V generation was demonstrated at 80 K. The enhancement mechanism was also investigated using soft and hard X-ray photoelectron spectroscopy (SXPES, HAXPES), and soft X-ray absorption spectroscopy with synchrotron radiation. This report reveals a way to new voltage source applications employing the BPVE for high impedance devices with ferroelectrics. Important aspects for designing ferroelectric materials by impurity doping are also discussed.

## Introduction

The bulk photovoltaic effect (BPVE)^1–5^ in ferroelectric materials has been intensively investigated because of properties such as above bandgap photovoltage generation or the possibility of high power conversion efficiency that exceeds the Shockley–Queisser limit^6^. One of the most highly anticipated applications that could take advantage of such high photovoltage is optical actuators using ferroelectrics that exhibit inverse piezoelectric properties^7–9^. The electric field generated by the BPVE in ferroelectrics is caused by strain due to the converse piezoelectric effect; therefore, optical strain could be induced by the coupling of these effects.

Optically induced strain in ferroelectrics has been previously investigated for BaTiO_3_^9^ and (Pb,La)(Zr,Ti)O_3_ (PLZT)^7,8^; however, some issues remain to be overcome. Firstly, the mechanism of the BPVE has not yet been completely clarified, although the piezoelectricity in ferroelectrics has been intensively investigated since the discovery of a morphotropic phase boundary (MPB) in Pb(Zr,Ti)O_3_ (PZT). Secondly, typical ferroelectrics such as BaTiO_3_ and PZT have a bandgap in the UV light region of > 3 eV^10,11^, making the desired visible light utilization difficult.

Regarding the first issue, a shift current has recently been discussed intensively as one of the mechanisms for the BPVE^12–17^. The shift current is a quantum mechanical effect that generates photocurrent, which has quite a different mechanism from that of p–n junction type solar cells. With respect to the photocurrent generated by the shift current, although it is responsive to unpolarized light, an even larger response is expected under polarized light. The ferroelectric polarization is also closely related to the shift current response, although it is not necessary for the generation of a shift current. A larger open circuit voltage (*V*_OC_) can also be obtained in a highly insulating medium.

Regarding the second issue, BiFeO_3_ (BFO) is an environmental-friendly, lead-free multiferroic that simultaneously exhibits excellent ferroelectric properties and antiferromagnetism^18,19^. The bandgap of BFO is in the visible wavelength region of 2.5–2.8 eV^20,21^, by which visible light driven devices can be realized. In addition, super-long-time relaxation of photo-induced influence have also reported^22^. Ultra-fast response and optical strain^23^ with its modulation by magnetic fields^24^ have already been reported for multiferroic BFO. However, the strain in BFO thin films can only be observed under irradiation by a femtosecond laser. Even bulk BFO single crystals show only a small strain of ca. 0.0002%. To enhance the optical strain, enhancement of the photovoltage is required; therefore, a highly insulating BFO thin film is necessary.

Here, we demonstrate a photovoltage above 852 V due to the BPVE in a lead-free multiferroic Mn-doped BiFeO_3_ (BFMO) single crystal thin film. We also clarify that evolution of the electronic structure by the Mn-doping of BFO has an important role in enhancement of the photovoltage.

## Results

### BFMO thin film growth

**Figure 1:**
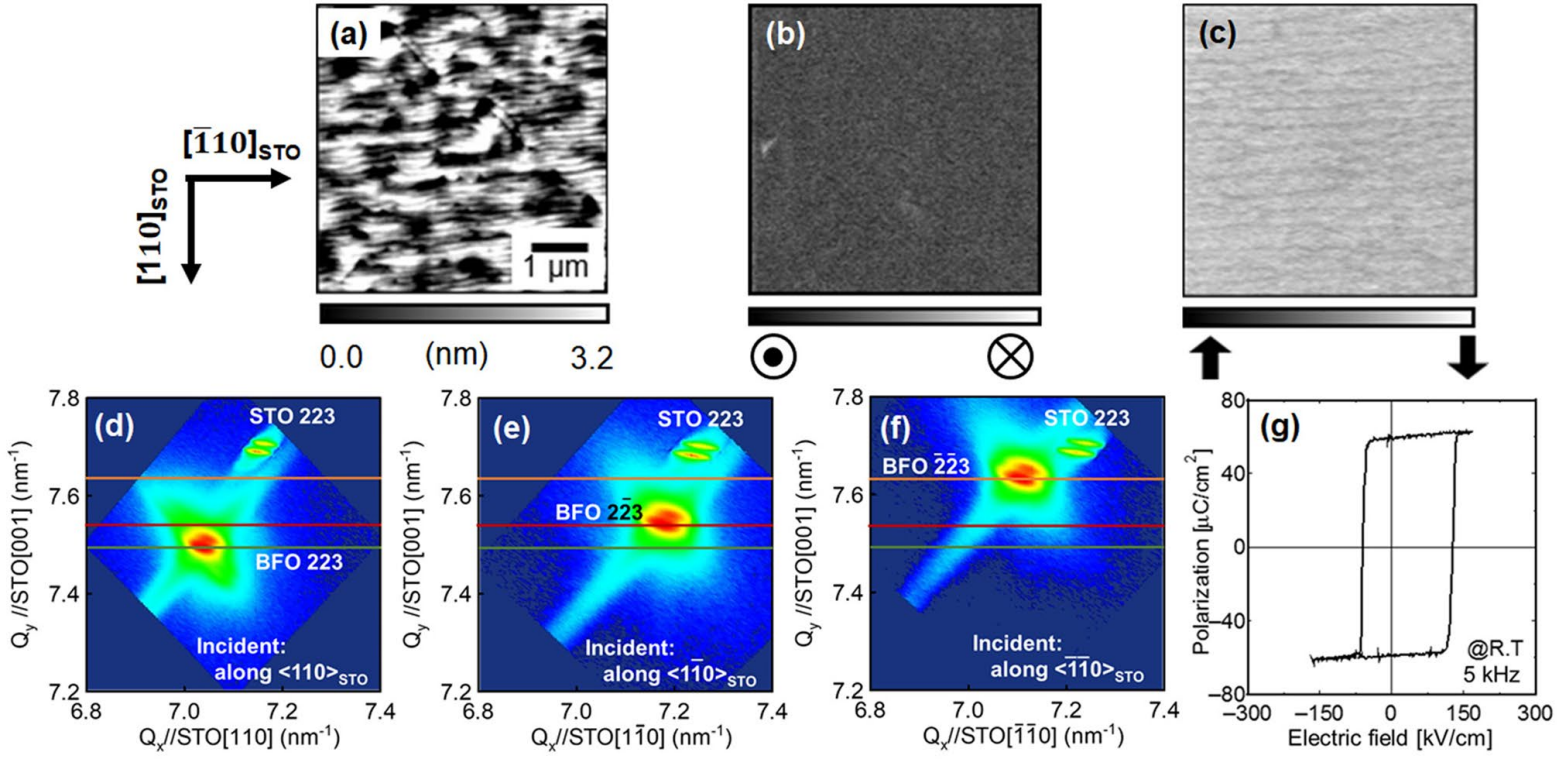
**a** Surface AFM image of 1 µm thick Mn 1 at%-doped BFO thin film grown on SRO-buffered STO, and **b** vertical- and **c** lateral-PFM images of the thin film. XRD-RSM around **d** BFMO 223, **e** BFMO 2$$\overline{2}$$3, and **f** BFMO $$\overline{2}$$$$\overline{2}$$3 diffraction spots. **g** Dielectric displacement–electric field hysteresis loop of the thin film measured at RT. Note that the BFMO thin film is a single crystal and shows an excellent ferroelectric *D–E* hysteresis loop.

To characterize the BPVE of BFMO thin films, a single crystal film is suitable because the geometric relation between the incident light polarization and the BFMO crystal has a strong effect on the photocurrent. In addition, the BPVE measurements were performed in the film plane, meaning that film orientation having lower azimuthal angle ***P***_***s***_ vector is suitable. Therefore, 1 µm thick BFMO thin films with various amounts of dopant (0, 0.5, 1.0, 3.0 and 10 at%) were grown on vicinal SrTiO_3_(001) (STO) substrates by conventional RF planar magnetron sputtering process. Vicinal STO substrates with the [001]_STO_ direction inclined 4° toward [110]_STO_ have been used for the domain engineering^25–28^ of BFMO thin films. Figure 1a–c show surface atomic force microscopy (AFM), and vertical and lateral piezoresponse force microscopy (PFM) images of a Mn 1 at%-doped BFO thin film. The BFMO thin film has a well-aligned step-and terrace structure with the step propagation direction along [110]_STO_, which indicates step-flow growth of the film. The vertical and lateral PFM images show uniform contrast, which reveals that the spontaneous polarization (***P***_**s**_**)** direction is aligned along the [111]_STO_ direction over the entire region.

For macroscopic characterization, X-ray diffraction reciprocal space mappings (XRD-RSMs) around BFO 223, $$\overline{2}$$23/2$$\overline{2}$$3 and $$\overline{2}$$$$\overline{2}$$3 diffraction spots were measured, as shown in Fig. 1d–f, respectively. All mappings show a single BFMO diffraction spot, except for the diffraction spots from the STO substrate. The doubled spots in each pattern are due to the mixed incidence of Cu Kα_1_ and Kα_2_ radiation. These BFMO {223} spots have three different d-spacings due to the rhombohedral symmetry. Furthermore, microscopic and macroscopic structural analyses reveal that the BFMO film is completely single crystal, and that 0, 0.5, and 3.0 at% Mn-doped BFMO thin films are also single crystal. Details of the domain structure of the BFMO thin films are described in the Supplementary Information.

To confirm the ferroelectricity of the BFMO thin films, ferroelectric *D-E* hysteresis loops of Pt/1 µm thick BFMO/SrRuO_3_ (SRO)/STO capacitor structures were measured. The capacitor with a 1 at% Mn-doped BFMO thin film shows well-saturated square shape *D-E* hysteresis loops at room temperature (RT), as shown in Fig. 1g. The double remanent polarization (2*P*_r_) and double coercive field (2*E*_C_) of the BFMO thin film were 120 µC/cm^2^ and 250 kV/cm, respectively. The 2*P*_r_ value agrees well with that for BFO bulk single crystal^20^. The other samples, except for the 10 at% Mn-doped BFMO thin film, also showed well-saturated square shape *D-E* hysteresis loops at RT (see Supplementary Information).

### BPVE in BFMO thin films

**Figure 2:**
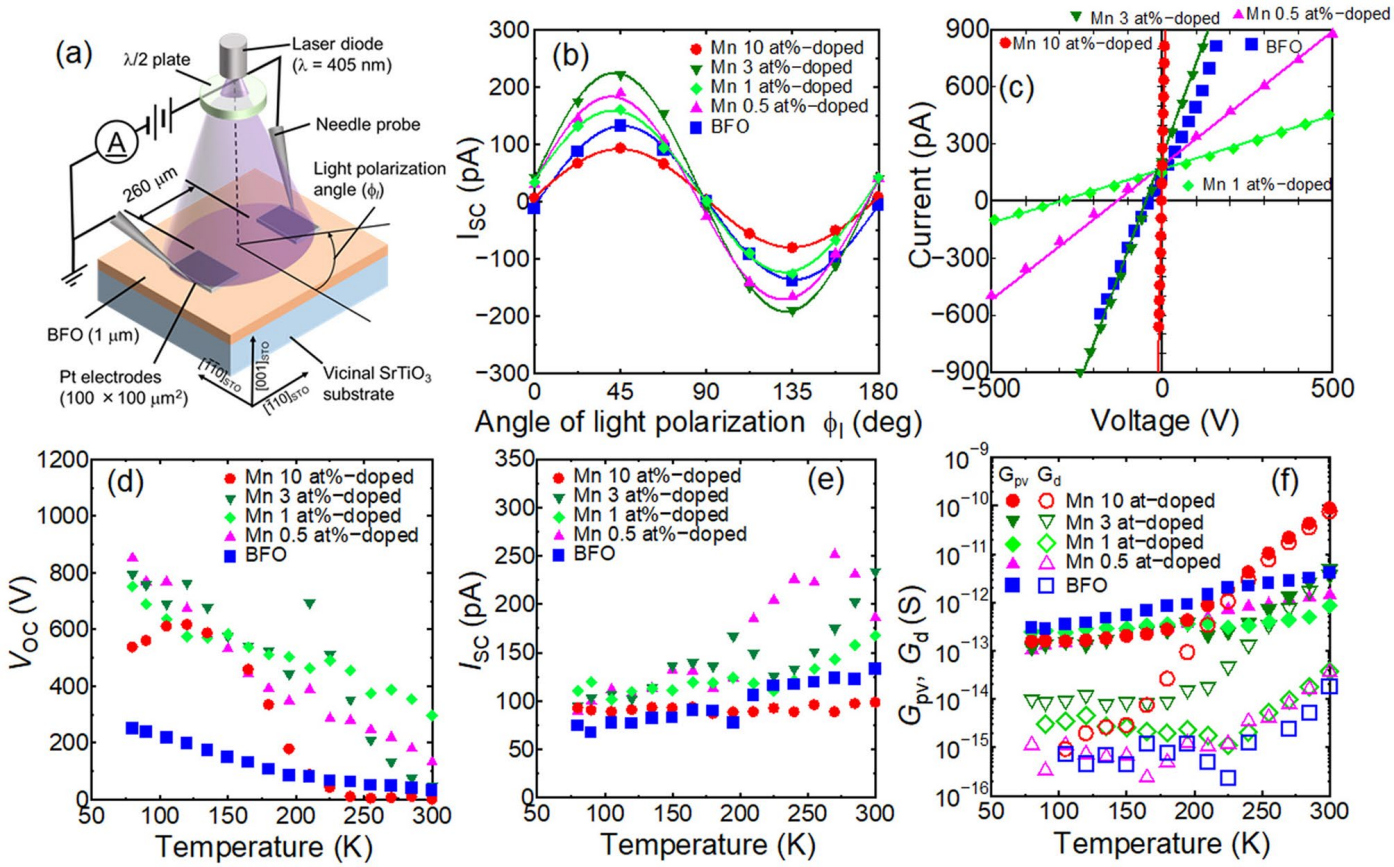
**a** Schematic illustration of the setup for photovoltaic property measurements. **b** Dependence of the light polarization angle on the short circuit current (*I*_SC_). **c** *I–V* characteristics, and temperature dependence of **d** *I*_SC_, **e** open circuit voltage (*V*_OC_), **f** photoconductance (*G*_pv_) and dark conductance (*G*_d_) for BFMO thin films with various amounts of Mn doping. The photovoltaic properties were measured under irradiation from a blue violet laser (λ = 405 nm). The light polarization angle (*ϕ*_l_) was fixed at 45° in **c**–**f**. Note that the maximum *V*_OC_ was 280 V at RT for Mn 1 at%-doped BFO, and was 852 V at 80 K in Mn-0.5 at%-doped BFO thin films.

To characterize the BFMO thin films, Pt/BFMO/Pt coplanar capacitors were fabricated on vicinal STO substrates, as shown in Fig. 2a. Pt electrodes (100 × 100 μm^2^) were fabricated on the BFMO surface along the [1$\overline{1}$0]_STO_ direction with an inter-electrode distance of 260 μm. The current–voltage (*I*–*V*) characteristics of the Pt/BFMO/Pt coplanar capacitors were measured at RT under irradiation from a blue violet laser (*λ* = 405 nm). For these measurements, light polarization was always kept in the film plane and the laser power density was set at 17 W/cm^2^ using a 60 mW laser diode.

Figure 2b shows the dependence of the light polarization angle (*ϕ*_l_) on the short circuit current (*I*_SC_) of the Pt/BFMO/Pt coplanar capacitors. Light polarization along the [100]_STO_ direction is represented by *ϕ*_l_ = 0°. The light polarization was then rotated by inserting a *λ*/2 plate. A positive *ϕ*_l_ indicates counterclockwise rotation of the light polarization. The *I*_SC_ values for the Pt/BFMO/Pt capacitors with various amounts of Mn dopant in the BFMO layer showed doubling sinusoidal changes, which indicates that the photocurrents are due to the BPVE.

In principle, the BPVE is a second-order nonlinear optical effect that includes carrier excitation processes. Therefore, the photocurrent ***J***_i_, can be given as

1 $${\varvec{J}}_{{\text{i}}} = I_{0} {\varvec{\beta}}_{{{\text{ijk}}}} {\varvec{e}}_{{\text{j}}} {\varvec{e}}_{{\text{k}}} ,$$

where *I*_0_ is the incident light intensity, ***β***_ijk_ is the bulk photovoltaic tensor of rank three, and ***e***_j_ and ***e***_k_ are components of the light polarization vectors of two photons. For BFO crystals belonging to the point group 3 m, Eq. 1 can be represented by

2 $$\left( {\begin{array}{*{20}c} {j_{1} } \\ {j_{2} } \\ {j_{3} } \\ \end{array} } \right) = I_{0} \left( {\begin{array}{*{20}c} 0 & 0 & 0 & 0 & {\beta_{15} } & { - \beta_{22} } \\ { - \beta_{22} } & {\beta_{22} } & 0 & {\beta_{15} } & 0 & 0 \\ {\beta_{31} } & {\beta_{31} } & {\beta_{33} } & 0 & 0 & 0 \\ \end{array} } \right) \left( {\begin{array}{*{20}c} {e_{1}^{2} } \\ {e_{2}^{2} } \\ {e_{3}^{2} } \\ {e_{2} e_{3} } \\ {e_{3} e_{1} } \\ {e_{1} e_{2} } \\ \end{array} } \right).$$

where the first index of the tensor components corresponds to the current direction and the second index is that used in Voigt reduced notation. The coordinates of the system are Cartesian coordinates {***x***_**i**_} with ${\varvec{x}}_{1} = \left( {\frac{a}{2}, - \frac{a}{2\sqrt 3 }, \frac{c}{3}} \right)$, ${\varvec{x}}_{2} = \left( {0, \frac{a}{\sqrt 3 }, \frac{c}{3}} \right)$, and $, {\varvec{x}}_{3} = \left( { - \frac{a}{2}, - \frac{a}{2\sqrt 3 }, \frac{c}{3}} \right)$, where *a* and *c* are the lattice constants of BFO with a hexagonal setting. From Eqs. 1 and 2, *I*_SC_ of the Pt/BFMO/Pt coplanar capacitor can be given by

3 $$I_{{{\text{SC}}}} \propto I_{0} \left( { - 0.41\beta_{15} - 0.574\beta_{22} } \right)\sin 2\phi_{{\text{l}}} .$$

This theoretical formula agrees well with the experimental results given in Fig. 2b. The maximum *I*_SC_ was determined to be *ϕ*_l_ = 45°, which is in good agreement with that for the BPVE in BFO-based materials^29–33^.

The enhancement of *I*_SC_ by Mn-doping of BFO was also confirmed. A maximum *I*_SC_ of 224 pA was confirmed for the Mn 3 at%-doped BFO thin film, which is known as gap-state engineering, as proposed by Matsuo et al.^34^ However, the lower *I*_SC_ for the Mn 10 at%-doped BFO thinilm indicated an increase in free carriers that absorb incident light.

Mn doping was determined to significantly change the *I–V* characteristics of the Pt/BFMO/Pt coplanar capacitors under light irradiation. From Fig. 2c, the *V*_OC_ increased significantly up to a Mn doping amount of 1 at%, and the maximum value reached 280 V, which corresponds to an electric field of 10.8 kV/cm. For doping over 1 at% Mn, *V*_OC_ decreased significantly. Theoretically, *V*_OC_ can be expressed by

4 $$V_{{{\text{OC}}}} = \frac{{I_{SC} d}}{{G_{{\text{d}}} + G_{{{\text{pv}}}} }} ,$$

where *d* is the inter-electrode distance, and *G*_d_ and *G*_pv_ are the conductance under dark and irradiation conditions, respectively. Equation 4 reveals that *V*_OC_ was enhanced by a decrease in the conductivity or an increase in *I*_SC_. According to the *I-V* characteristics shown in Fig. 2c, although *I*_SC_ did not change significantly, the conductance did vary significantly due to Mn-doping. Therefore, the enhancement of *V*_OC_ is due to a decrease of the photoconductance in the BFMO thin films.

Notably, *V*_OC_ was significantly enhanced at low temperature. In particular, *V*_OC_ for Mn-doped BFO was significantly increased, as shown in Fig. 2d. A maximum *V*_OC_ of 852 V was obtained for Mn 0.5 at%-doped BFO at 80 K, which corresponds to an electric field of 32.8 kV/cm. Details of the photovoltaic properties at 80 K are described in the Supplementary Information. The drastic enhancement of *V*_OC_ was also due to the decrease of conductance under dark and irradiation conditions. *I*_SC_ as shown in Fig. 2e was not so significantly increased; however, the dark- and photoconductance were significantly decreased with the temperature, as shown in Fig. 2f. As a result, the enhancement of *V*_OC_ in Mn-doped BFO thin films is considered to be caused by an enhancement of the insulation properties. Since the discovery of ferroelectricity in BFO thin films, Mn doping of BFO has been intensively investigated to decrease the leakage current of BFO thin films; however, the influence of Mn doping on the electronic structure remains unclear.

### Electronic structure of Mn-doped BFO thin films

**Figure 3:**
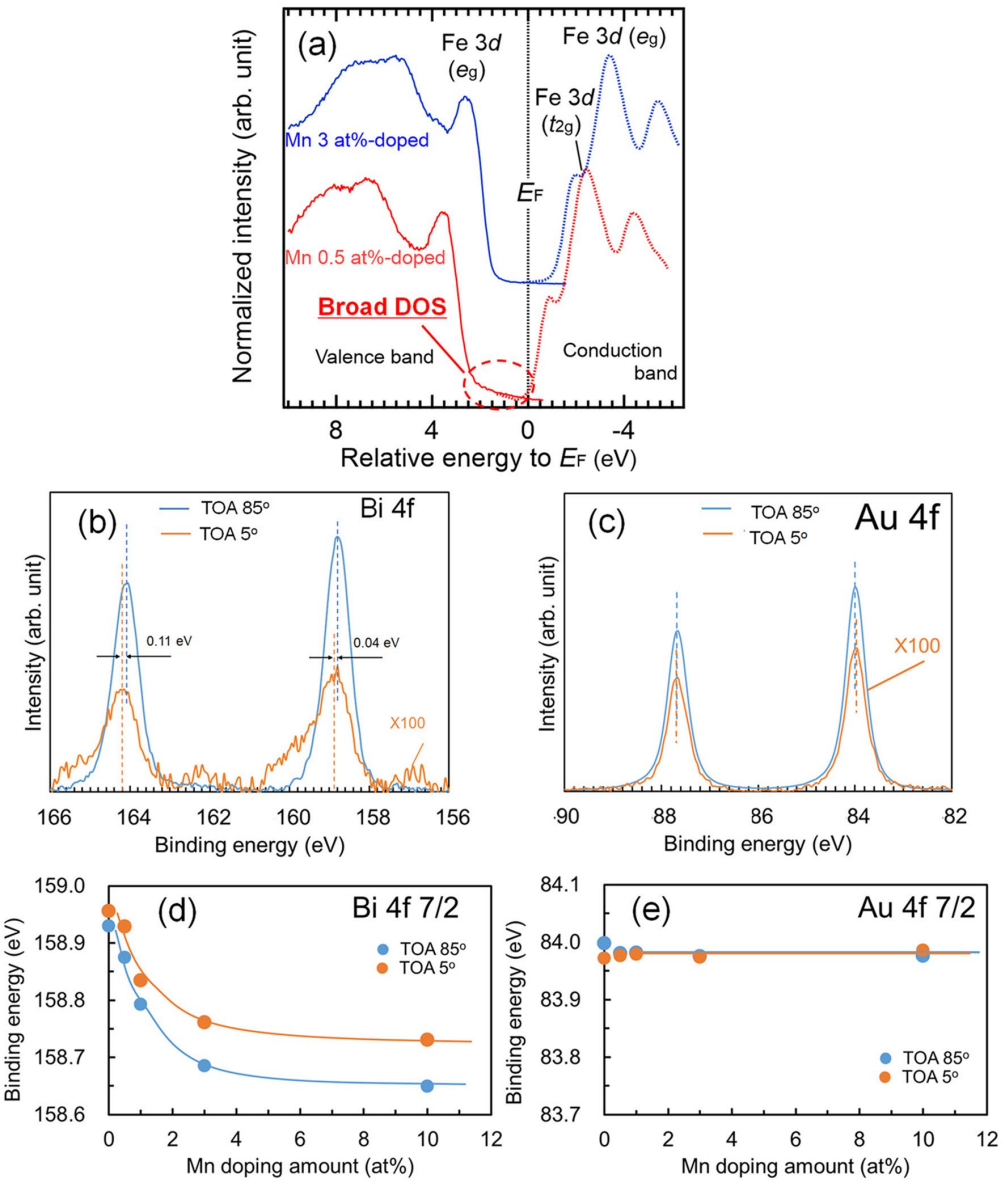
**a** Valence and conduction band spectra for Mn 0.5 at%-doped (blue line) and 3 (red line) at%-doped BFO thin films measured using SXPES and SXAS, HAXPES spectra of **b** Bi 4f and **c** Au 4f orbitals measured at TOAs of 5° (orange line) and 85° (blue line). Dependence of **d** Bi 4f_7/2_ and **e** Au 4f_7/2_ binding energies measured at TOA of 5° (orange line) and 85° (blue line) on the amount of Mn doping. The broad DOS above the valence band is denoted by a red-dashed line in **a** and was observed in the Mn 0.5 at%-doped BFO thin film but vanished in the Mn-3 at%-doped BFO thin film.

To clarify the influence of Mn-doping in BFO on the electronic structure, soft X-ray photoemission spectroscopy (SXPES) and soft X-ray absorption spectroscopy (SXAS) using synchrotron radiation was performed on 0.5 and 3 at% Mn-doped BFO thin films at RT. 150 nm thick single crystal BFMO thin films were fabricated on SrRuO_3_-buffered vicinal STO(001) single crystal substrates. For preventing from charging up during SXPES and SXAS spectra, the maximum thickness is 150 nm. According to our previous study^28^, epitaxial strains of 150 nm and 1 μm thick BFO thin films can be estimated as -0.45% and − 0.30%, respectively, indicating small difference. In addition, the first principles calculations predicted that the epitaxial strains mainly cause symmetrical change of the electronic structure^36^. Moreover, it has already proved experimentally that the BFO thin films with various film thickness show constant bandgap^37^. Therefore, influence of the Mn doping on the valence band and conduction band spectra of the BFO thin films can be evaluated by using 150 nm thick films. Figure 3a shows the valence and conduction band spectra of the BFMO thin films. The 3 at% Mn-doped BFO thin film has a slightly smaller bandgap (*E*_g_) of ca. 2.0 eV compared to that of the 0.5 at% Mn-doped BFO at ca. 2.3 eV. However, a broad density of states (DOS) was observed above the valence band in the 0.5 at% Mn-doped BFO thin film, which caused a Fermi level shift of ca. 1.1 eV. O 1 s Fe 2p SXPES spectra also shifted by ca. 1.1 eV due to the Mn-doping of BFO (see Supplementary Information).

SXPES is a very powerful method for the investigation of the electronic structure of a material surface; however, characterization of a ferroelectric surface is very difficult due to polarization charges. Spontaneous polarization charges at the surface of a ferroelectric are generally terminated by molecules adsorbed from the air. However, the surface potential is unstable because of its floating potential. Therefore, the surface potential of the ferroelectrics in an ultrahigh vacuum should be more unstable due to unstable termination of the spontaneous polarization charges. Therefore, the SXPES and SXAS spectral shift with ferroelectrics cannot be completely elucidated.

Hard X-ray photoemission spectroscopy (HAXPES) of ultrathin metal film-capped surfaces is suitable for the precise evaluation of Fermi level shifts in ferroelectrics. The ferroelectric polarization charge is terminated by ultrathin metal films, which indicates that the Fermi level of the ferroelectric at the metal/ferroelectrics interface is pinned by that of the metal. HAXPES can also be used to evaluate a ferroelectric layer through the capped ultrathin metal film due to the deep penetration depth (> 20 nm) of high energy photoelectron.

Figures 3b,c show Au 4f and Bi 4f HAXPES spectra, respectively, for the Au (9 nm) capped Mn 1 at%-doped BFO/SRO/STO structure measured using 8 keV synchrotron X-rays with take-off angles (TOAs) of 85° (TOA85) and 5° (TOA5). The low and high TOAs mean surface and bulk sensitive conditions, respectively. The Bi 4f spectra with a TOA of 5° is slightly shifted to lower binding energy compared to those with TOA of 85°, which reveals that the energy bands of BFMO were bent up near the Au/BFMO interface. Figure 3d shows a larger shift of the Bi 4f spectra with an increase in the amount of Mn doping. The difference between the binding energy of Bi 4f spectra with TOAs of 85° and 5° also increased clearly with the amount of Mn doping. With an almost constant binding energy of the Au 4f spectra, as shown in Fig. 3e, the Fermi level in the BFMO decreased with an increase in the amount of Mn doping.

## Discussion

From Fig. 2, *V*_OC_ above the bandgap is due to the BPVE. Bhatnagar et al.^35^ already reported an above-bandgap *V*_OC_ of ca. 55 V at 80 K due to the BPVE and domain wall photovoltaic effect. The *V*_OC_ corresponds to an electric field of 5.5 kV/cm. On the other hand, *V*_OC_ in non-doped BFO at 80 K in the present study was 251 V, which corresponds to an electric field of 9.7 kV/cm. When the difference of film thickness between the present study and the previous study is considered, the *V*_OC_ of non-doped BFO in Fig. 2 was in agreement with that in the previous study. In contrast, the *V*_OC_ of 852 V for Mn 0.5 at%-doped BFO indicates a significant enhancement. According to Eq. (4), *V*_OC_ is a function of *I*_SC_, *G*_d_ and *G*_pv_. However, *I*_SC_ did not change significantly by Mn doping or by variation of the temperature when compared to the *V*_OC_ enhancement shown in Fig. 2c. The low temperature dependence of *I*_SC_ is an important feature of the BPVE, which indicates that *I*_SC_ is independent of the carrier mobility. In addition, *G*_pv_ was sufficiently larger than *G*_d_, as shown in Fig. 2f; therefore, the enhancement of *V*_OC_ was mainly caused by the decrease of *G*_pv_.

An improvement of insulation by the Mn doping of BFO has been investigated by many researchers^38–40^, although the mechanism of this improvement remains unclear. However, Noguchi et al.^41^ recently predicted by first principles calculations that trapping oxygen vacancies at the nearest O site of a doped Mn atom causes the impurity state above the valence band to disappear, which indicates an improvement of the insulation of Mn-doped BFO. A broad DOS is clearly evident in Mn 0.5 at%-doped BFO, as indicated by the red-dashed circle in Fig. 3a. In contrast, the broad DOS vanishes in Mn 3 at%-doped BFO. These results are in good agreement with the prediction reported by Noguchi et al*.*^39^, which indicates that these results experimentally confirm that oxygen vacancies trapped around doped-Mn atoms improve the insulation of Mn-doped BFO.

**Figure 4:**
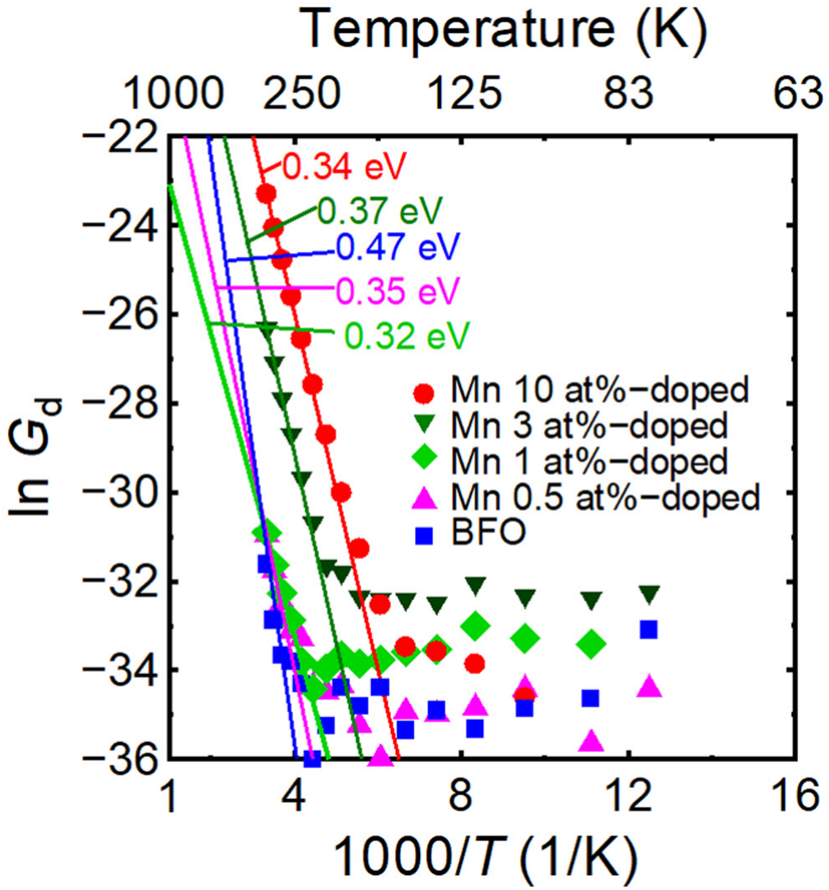
Arrhenius plot of dark conductance (*G*_*d*_) for BFMO thin films with various amounts of Mn doping. The values in the figure denote the slopes of the lines, which indicate activation energies. Note that the significant increase of *G*_d_ near RT in Mn-doped BFO is due to traps with a depth around 0.35 eV.

In addition, Fig. 3 shows that the Mn doping of BFO is acceptor doping, which indicates the introduction of impurity states. To evaluate these impurity states, *G*_d_ shown in Fig. 2e was replotted on an Arrhenius plot, as shown in Fig. 4. The slopes in Fig. 4 indicate the activation energy of the excited carriers. The activation energy can be evaluated to be ca. 0.35 eV in the BFMO thin films, despite a larger activation energy in non-doped BFO thin film. Therefore, the Fermi level shift as shown in Fig. 3 is due to these impurity states. Matsuo et al*.*^34^ have already reported that Mn doping of BFO creates half-occupied states ca. 1 eV from the valence band edge, from first principles calculations, considering a Mn valence of 3 + . This impurity state is slightly different from our experimental result of 0.35 eV. However, assuming a Mn impurity state that is 0.35 eV from valence band edge, the half-occupied state is filled by thermally excited electrons, as shown in the Arrhenius plot of Fig. 4; therefore, the Mn valence should be 2 + at RT and Mn doping could act as acceptor doping.

In conclusion, Mn-doping of BFO enhanced the photovoltage induced by the BPVE. In a Pt/Mn 1 at%-doped BFO/Pt coplanar capacitor with an inter-electrode distance of 260 μm, *V*_OC_ reached 280 V at RT (852 V at 80 K). This enhancement is considered to be due to improvement of the insulation of BFMO. Mn doping of BFO causes lowering of the Fermi level; however, the improvement of insulation was due to the disappearance of impurity states above the bandgap, which is an oxygen vacancy trapping effect around Mn dopant atoms. The high voltage generation realizes the application of a high electric field in the BFMO thin film itself. Thus, we have discovered a new voltage source application for high impedance devices using ferroelectrics. Moreover, we presented experimental evidence for the influence of impurity doping of ferroelectrics on the electronic structure and electrical properties. These results provide important aspects for control of the conductivity of ferroelectrics by impurity doping.

## Methods

### Sample preparation

Vicinal STO(001) single crystals were used as substrates for all samples. A Ti–O terminated surface was formed by wet etching with buffered-HF solution and annealing at 1,000 °C for 1 h in the atmosphere. For samples for SXPES, SXAS, and HAXPES measurements, 30 nm thick SRO thin film was epitaxially grown on the vicinal STO surface by an RF planar magnetron sputtering process. For SRO thin film growth, a 2 inch diameter SRO ceramic was used as a target. The substrate temperature, sputtering pressure, Ar/O_2_ flow rate ratio, and RF power were fixed at 580 °C, 13 Pa, 9.5 sccm/0.5 sccm, and 30 W, respectively. For photovoltaic property measurements, 1 μm thick BFMO thin films were directly grown on vicinal STO substrates by an RF planar magnetron sputtering process. For BFMO growth, the substrate temperature, sputtering pressure, Ar/O_2_ flow rate ratio, and RF power were fixed at 655 °C, 0.5 Pa, 3.5 sccm/1.5 sccm, and 50 W, respectively. Bi_2_O_3_, *α*-Fe_2_O_3_, and Mn_2_O_3_ powders were mixed and calcined with Bi/Fe/Mn composition ratios of 1.05/1−*x*/*x* (*x* = 0, 0.005, 0.01, 0.03 and 0.1), pressed into 4 inch diameter pellets and used as a target. For SXPES, SXAS, and HAXPES measurements, 150 nm thick BFMO thin films were grown on SRO-buffered vicinal STO substrates by an RF planar magnetron sputtering process with the conditions for BFMO growth described above. The BFMO thin films were also grown on dummy STO(001) substrates at the same lot for checking film thickness by cross-sectional SEM images. For measurement of the photovoltaic properties, 100 × 100 μm^2^ Pt electrodes were deposited on the BFMO/STO surface by RF planar magnetron sputtering and a photolithography lift-off processes. For HAXPES measurements, 9 nm thick ultrathin Au films were deposited on the BFMO/SRO/STO surface by thermal evaporation.

### SXPES and SXAS measurements

SXPES and SXAS measurements were performed at the BL-2A MUSASHI undulator beamline at the Photon Factory of The High Energy Accelerator Research Organization (KEK), Tsukuba, Japan. The SXAS spectra were recorded in total electron yield mode. The SXPES spectra were acquired using a VG-Scienta SES-2002 hemispherical analyzer. The SXPES and SXAS resolutions were set at approximately 100 and 80 meV, respectively.

### HAXPES measurements

The HAXPES measurements were performed at the BL47XU at the SPring-8 synchrotron radiation facility, Sayo, Japan. Synchrotron radiation X-rays with an energy of 7.94 keV were used for photoelectron excitation. Bi 4f, Au 4f and valence band spectra were measured at RT and peak positions were corrected with respect to the Fermi edge of a gold film sample. The HAXPES spectra were obtained using the R4000 photoelectron analyzer (VG-Scienta Co.) equipped with a wide-acceptance-angle electrostatic lens with acceptance angle of about ± 32°^42,43^. Both the top Au-capped layer and SRO bottom electrodes were grounded simultaneously during the measurements.

## Supplementary information

Supplementary Information.

## Data Availability

The datasets analyzed during the current study are available from the corresponding author on reasonable request.
