# Supplementary material for: Enhancement of photovoltage by electronic structure evolution in multiferroic Mn-doped BiFeO3 thin films
Source: Sci Rep. 2020 Sep 21;10:15108. doi: 10.1038/s41598-020-71928-5 (PMC7505956; doi:10.1038/s41598-020-71928-5)
Supplement: Supplementary file 1 — Supplementary Information. [file 41598_2020_71928_MOESM1_ESM.pdf]

Supplementary Information

Enhancement of Photovoltage by Electronic Structure Evolution in Multiferroic Mn-doped $BiFeO_3$ Thin Films

Seiji Nakashima,[1,a)] Tohru Higuchi,[2] Akira Yasui,[3] Toyohiko Kinoshita,[3], Masaru Shimizu,[1] & Hironori Fujisawa[1]

[1] *Department of Electronics and Computer Sciences, Graduate School of Engineering, University of Hyogo, Himeji, Hyogo 671-2201, Japan*
[2] *Department of Applied Physics, Tokyo University of Science, Katsushika, Tokyo 125-8585, Japan*
[3] *Japan Synchrotron Radiation Research Institute / SPring-8, Sayo, Hyogo 679-5148, Japan*

[a)] nakashima@eng.u-hyogo.ac.jp

Domain structure of Mn-doped $BiFeO_3$ thin films

Figure S1 shows AFM, vertical- and lateral-PFM images of 1 μm thick Mn-doped $BiFeO_3$ (BFOM) thin films with Mn doping amounts of 0, 0.5, 3, and 10 at%, respectively. The vertical-PFM images of all BFMO thin films show a black contrast in the entire region, which indicates that $\boldsymbol{P}_s$ vectors have components along $[001]_{STO}$. In addition, lateral-PFM images show white contrast in the entire region, except for the Mn 10 at%-doped BFO thin film, which shows white and grey regions. Therefore, the BFMO thin films with Mn doping amounts of 0, 0.5, 1, and 3 at% are single domain films with $\boldsymbol{P}_s$ vectors along $[111]_{STO}$. In contrast, the Mn 10 at%-doped BFO thin film shows white and grey contrast in the lateral PFM image and has a 71° domain structure with $[110]_{BFO}$//$[110]_{STO}$, $[\bar{1}10]_{BFO}$//$[110]_{STO}$ and $[1\bar{1}0]_{BFO}$//$[110]_{STO}$.

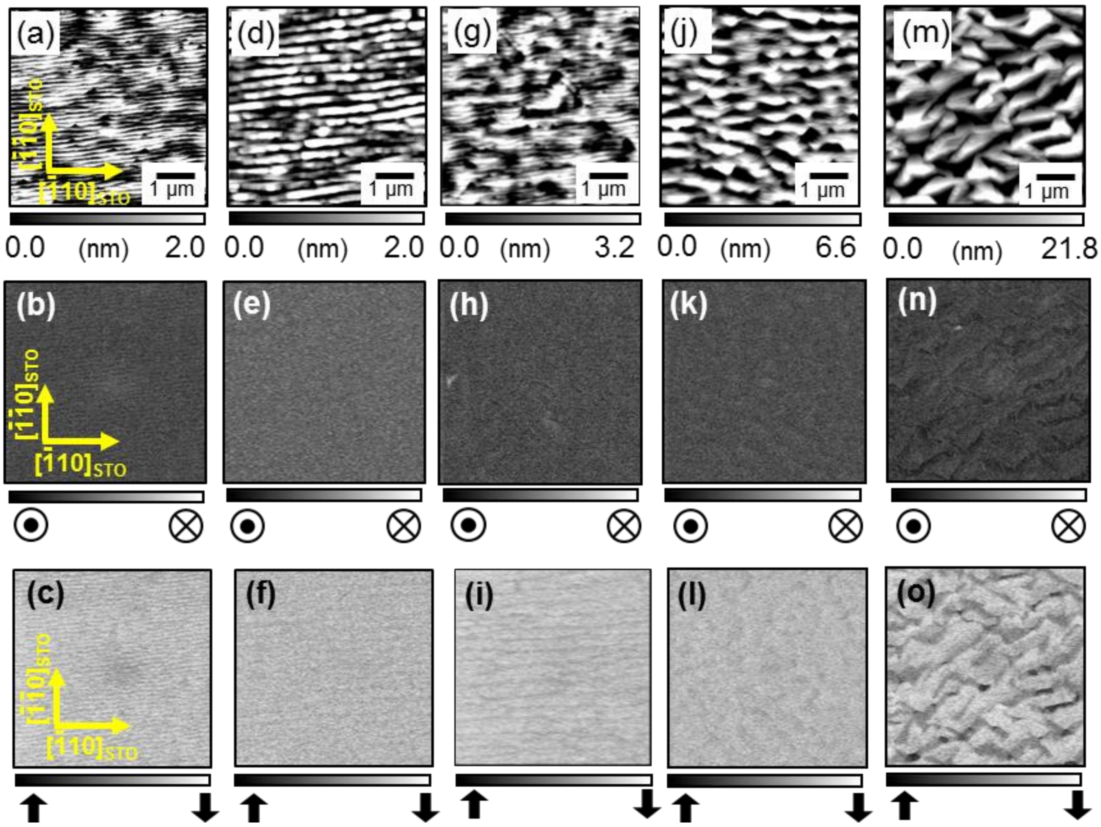

Figure S1 (a,d,g,j,m) Surface AFM, (b,e,h,k,n) vertical-PFM, and (c,f,i,l,o) lateral-PFM images of BFMO thin films with Mn doping amounts of (a-c) 0, (d-f) 0.5, (g-i) 1, (j-l) 3, and (m-o) 10 at%.

Crystallinity of Mn-doped $BiFeO_3$ thin films

Figure S2 shows XRD-RSMs around BFMO 004 diffraction spot of the BFMO thin films with Mn doping amounts of 0, 0.5, 1, 3 and 10 at%. Excepting for the Mn 10-at%-doped BFO thin film, the BFMO thin films show a single BFO 004 diffraction spot, indicating that these BFMO thin films are completely single domain. In contrast, only the Mn 10-at%-doped BFO thin film shows three diffraction spots, revealing that the BFMO thin film is multi domain. These results are in good agreement with the PFM images in Fig. S1. Moreover, the unit cell volume of BFMO thon film increases with increasing Mn doping mount, resulting the BFMO 004 diffraction spot slightly shifts toward a lower Q_y. For qualifying the crystallinity, full width of half maximums (FWHM) of ω rocking curve of the BFMO 004 diffraction spot in these BFMO thin films are summarized in Table S1. With increasing Mn doping amount, the FWHM of the BFMO 004 diffraction spot slightly increase, however, the values are from 0.10 to 0.19 deg, indicating these BFMO thin films have high crystallinity.

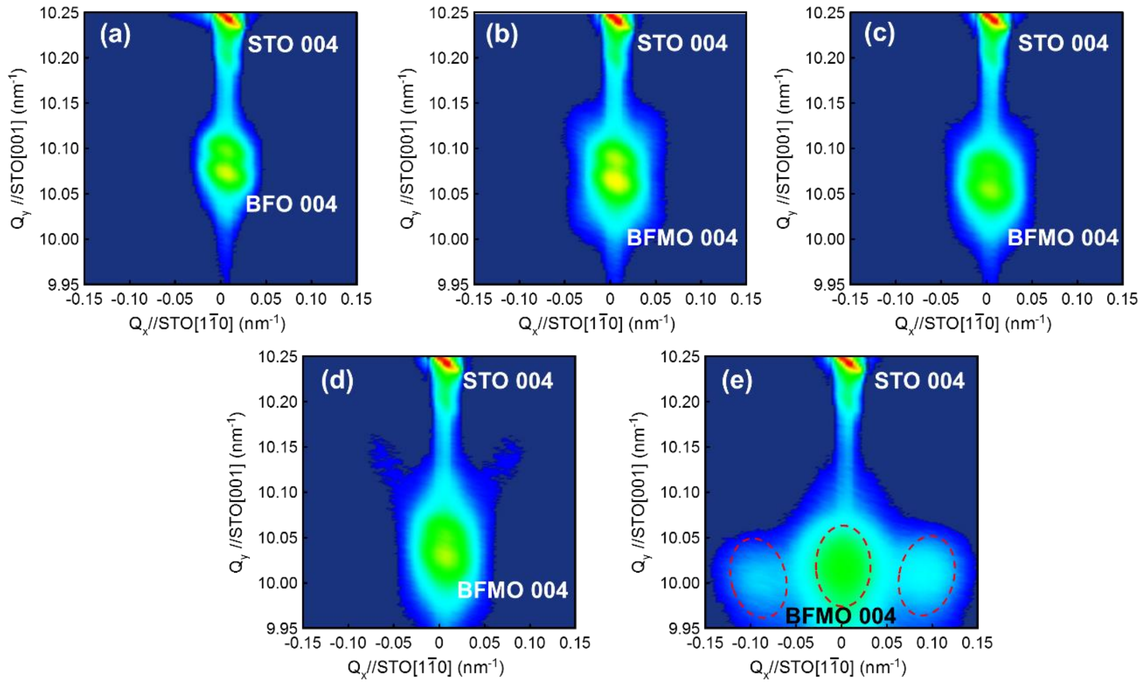

Figure S2 X-ray diffraction reciprocal space mappings around BFMO 004 diffraction spot of BFMO thin films with Mn doping amounts of (a) 0, (b) 0.5, (c) 1, (d) 3, and (e) 10 at%.

Table S1 Full width at half maximum of ω rocking curve of BFMO 004 diffraction peak in BFMO thin films with various Mn doping amount.

Mn doping amount (at%)	FWHM of ω rocking curve of BFMO 004 diffraction peak (deg)
0	0.12
0.5	0.10
1.0	0.15
3.0	0.16
10	0.19

Ferroelectric *D-E* hysteresis loops of Mn-doped $BiFeO_3$ thin films

Figure S3 show *D-E* hysteresis loops of Pt/300 nm thick BFMO/$SrRuO_3$/STO capacitors with Mn doping amounts of 0.5, 1, and 3 at% measured at RT. These BFMO thin films show well-saturated square shape *D-E* hysteresis loops at RT. The BFO thin film also shows a well-saturated *D-E* hysteresis loop, as described in *Jpn. J. Appl. Phys.* **52** 09KB03 (2013). The remanent polarization (P_r) values are approximately constant at 60 μC/cm^2, regardless of the amount of Mn doping. These results are in good agreement with that for BFO single crystal. The coercive field (E_c) increases from 90 to 170 kV/cm with the amount of Mn doping. No *D-E* hysteresis loop was observed for the Mn 10 at%-doped BFO thin film due to its high conductivity.

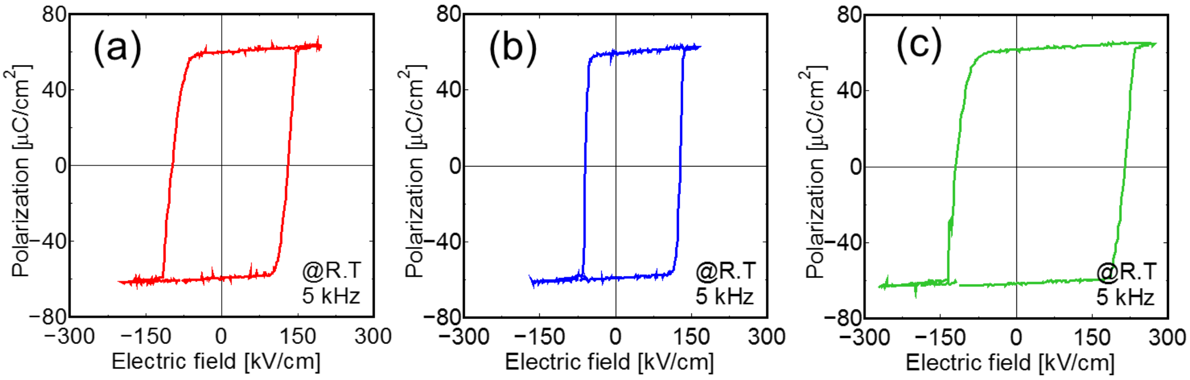

Figure S3. Ferroelectric *D-E* hysteresis loops for Pt/300 nm thick BFMO/SRO/STO capacitors with Mn doping amounts of (a) 0.5, (b) 1, and (c) 3 at%.

Photovoltaic properties of Mn 0.5 at%-doped $BiFeO_3$ thin film at 80 K

A Pt/Mn 0.5 at%-doped BFO/Pt coplanar capacitor shows a maximum open circuit voltage (V_{OC}) of 852 V at 80 K. A schematic illustration of the measurement geometry is shown in Fig. 2(a) of the main text. According to the BPVE, V_{OC} also sinusoidally changes as a function of twice the polarization angle ($2\phi_l$). Figure S4(a) shows *I*-*V* characteristics under irradiation from a blue violet laser (λ = 405 nm) with ϕ_l of 45°, 0° and 135°. Maximum and minimum V_{OC} of 852 V and -847 V were obtained at ϕ_l of 45° and 135°, respectively. The sinusoidal change of V_{OC} was confirmed, as shown in Fig. S4(b); therefore, V_{OC} can be continuously controlled from -852 to 854 V by light polarization. V_{OC} unlimited by bandgap is thus due to the BPVE.

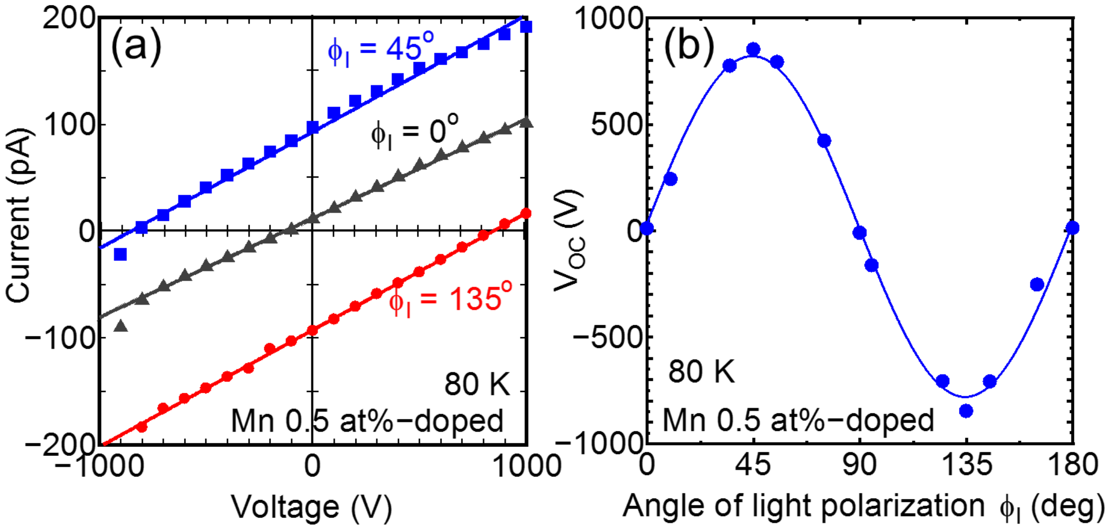

Figure S4. (a) *I*-*V* characteristics for a Pt/Mn 0.5-at%-doped BFO/Pt coplanar capacitor under irradiation from a blue violet laser at 80 K with a ϕ_l = 45°, 0°, and 135°, and (b) ϕ_l dependence of V_{OC}.

Fe 2p and O 1s SXPES spectra of Mn-doped $BiFeO_3$ thin film

Figure S5 shows Fe 2p and O 1s SXPES spectra of Mn 0.5 and 3 at%-doped BFO thin films. Both Fe 2p and O 1s SXPES spectra of the Mn 3 at%-doped BFO thin film were shifted ca. 1.1 eV to a lower binding energy compared to those of the Mn 3 at%-doped BFO thin film. Note that the broad peaks in the O 1s spectra are due to O-H bonds at the BFMO surface. These

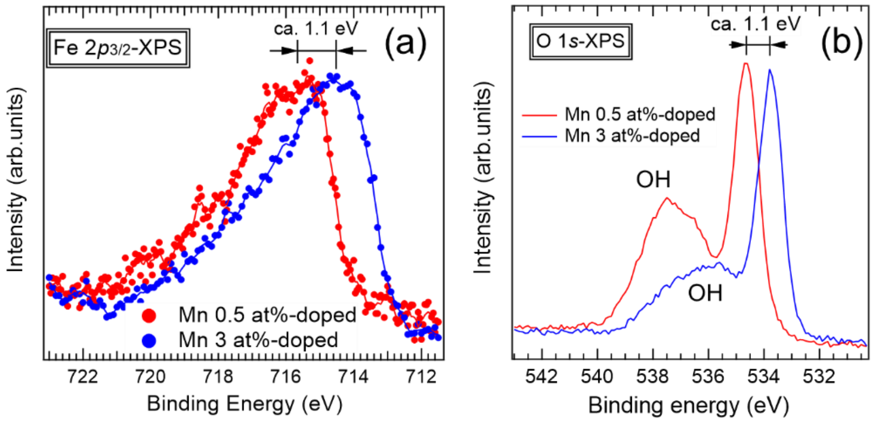

Figure S5. (a) Fe 2p and (b) O 1s SXPES spectra for Mn 0.5- and 3 at%-doped BFO thin films.

results confirm the Fermi level shift caused by Mn doping, as described in the main text.
